# Borges and the art of forgetting

**DOI:** 10.1192/j.eurpsy.2021.1995

**Published:** 2021-08-13

**Authors:** N. Ohri, A. Gill, M. Saini

**Affiliations:** 1 Psychiatry, New Life Hospital, Varanasi, India; 2 Psychiatry, New Life Hospital, Varanasi, India; 3 Psychiatry, Veer Chandra Singh Garhwali Govt. Institute of Medical Science & Research, Srinagar, India

**Keywords:** Jorge Luis Borges, literary work review, Hyperthymestic Syndrome, Autobiographical Memory

## Abstract

**Introduction:**

In 2005 Elizabeth Parker and fellow researchers described the first case of Hyperthymestic Syndrome, a woman going by initials AJ. Thereafter, a handful more of such cases have emerged. Older descriptions of extraordinary memory in medical literature mainly considered semantic and working memories. Jorge Luis Borges in his 1930s short story ‘Funes, his Memory’ writes about his, presumably fictitious, encounter with a man named Ireno Funes who possessed an extraordinary memory and a knack for keeping track of briefest of passing moment. Among many qualities that Funes and AJ share are their extraordinary memories, obsession for keeping track of time, and their problems with abstraction. After describing his extraordinary memory, Borges says of Funes, ‘I suspect nevertheless, that he was not very good at thinking. To think is to ignore (or forget) differences, to generalize, to abstract.’ Similarly, AJ has been described to have impaired abstraction, hypothesis formation and conceptual shifting. Moreover, both Funes and AJ see their capability as a burden rather than a gift. “My memory, sir, is like a garbage heap.” Says Funes.

**Objectives:**

A brief exploration of Jorge Luis Borges’ works in the context of autobiographical memory.

**Methods:**

The comparisons between Borges’ description of his character’s autobiographical memory and findings of modern research techniques will be done qualitatively.

**Results:**

Effort is made to undersatnd Borges philosophy in context of mordern memory research.

**Conclusions:**

An in depth look into Borges’ philosohies linking perception of time, coding of memory, abstration and language can inform further line of research regarding autobiographical memory.

**Disclosure:**

No significant relationships.

